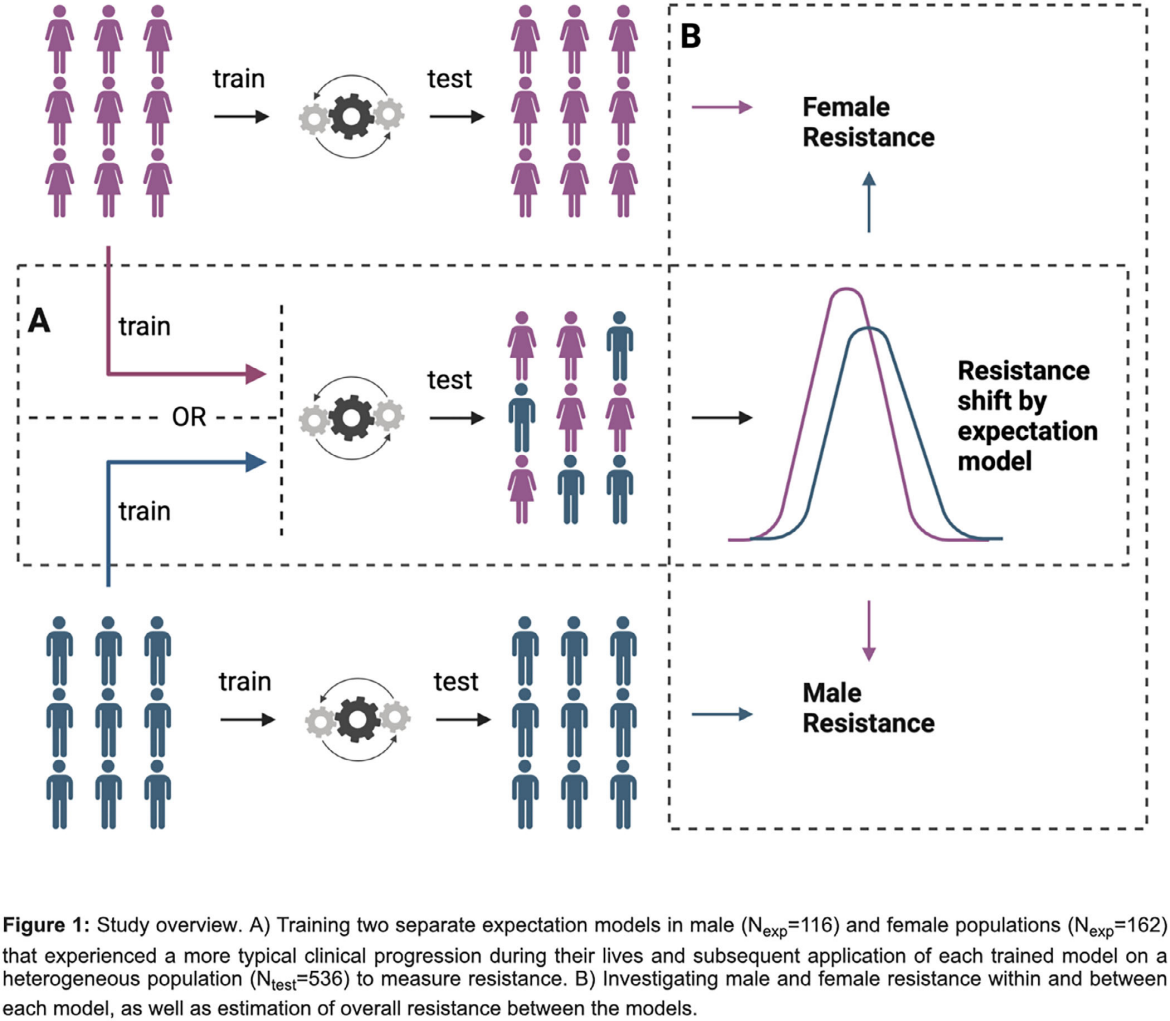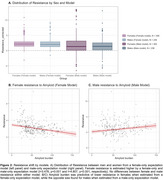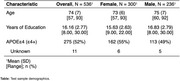# Sex differences in predictors of pathological resistance to cauTAUstrophe

**DOI:** 10.1002/alz70856_105762

**Published:** 2026-01-09

**Authors:** Maria Carrigan, Colin Birkenbihl, Hannah M Klinger, Oliver Langford, Gillian T Coughlan, Mabel Seto, Jane A Brown, Annie Li, Madison Cuppels, Michael J. Properzi, Jasmeer P. Chhatwal, Julie C Price, Aaron P. Schultz, Dorene M. Rentz, Rebecca E. Amariglio, Harm J. Krugers, Rik Ossenkoppele, Keith A. Johnson, Reisa A. Sperling, Timothy J. Hohman, Michael C. Donohue, Rachel F. Buckley

**Affiliations:** ^1^ Massachusetts General Hospital, Harvard Medical School, Boston, MA, USA; ^2^ Alzheimer Center Amsterdam, Department of Neurology, Amsterdam Neuroscience, Vrije Universiteit Amsterdam, Amsterdam UMC, Amsterdam, North Holland, Netherlands; ^3^ Faculty of Science, Swammerdam Institute for Life Sciences, University of Amsterdam, Amsterdam, North Holland, Netherlands; ^4^ Alzheimer's Therapeutic Research Institute, University of Southern California, San Diego, CA, USA; ^5^ Brigham and Women's Hospital, Harvard Medical School, Boston, MA, USA; ^6^ Clinical Memory Research Unit, Lund University, Lund, Sweden; ^7^ Vanderbilt Memory and Alzheimer's Center, Vanderbilt University School of Medicine, Nashville, TN, USA

## Abstract

**Background:**

As β‐amyloid (Aβ) accumulates in the brain, the prevailing theory posits that accelerating tau deposition escapes medial temporal (MTL) regions and encroaches upon neocortical areas. Evidence suggests some older adults demonstrate pathological resistance to caTAUstrophe (lower than expected levels of neocortical tau despite abnormal levels of Aβ). Given consistent evidence of higher tau levels in women than men, the aim of this study was to examine sex differences in the operationalization and predictors associated with resistance to neocortical tau burden in the context of Aβ proteinopathy.

**Method:**

Employing data from 814 Aβ+ older adults (N_Test_=536;Table) from three cohorts; A4/LEARN, ADNI and HABS. We calculated tau resistance using our published inverse learning method, which estimates the deviation from a model trained on an expectation sample (here, Aβ‐PET+ older adults with high neocortical tau‐PET burden[N_Exp_=278];Figure 1). For this study, the expectation samples were split into males‐only(*n* = 116) and females‐only(*n* = 162) to investigate how estimation of resistance for both sexes differed depending on the expectation sample. We then assessed the extent to which different factors (age, education, hippocampal volume, medial temporal tau‐PET, Aβ‐PET, CDR, PACC) differ by sex to predict tau resistance depending on the sex‐specific expectation (male‐only/female‐only) sample.

**Result:**

Despite no sex differences in MTL or neocortical tau‐PET, age, education or APOEe4 status in our training dataset (i.e., those representing caTAUstrophy), we found a shift of higher tau resistance estimation for both sexes(*p* <0.001) when trained on a female‐only sample(Figure 2A). Higher Aβ‐PET burden was associated with lower tau resistance in women from a female‐only training sample(Figure 2B), while lower Aβ‐PET burden was associated with lower tau resistance in men from a male‐only sample(Figure 2C). Global CDR(*p* <0.001), education(*p* <0.05), and ICV‐adjusted hippocampal volume(*p* <0.001) were found to significantly predict male tau resistance estimated from a male‐only training sample but not for female tau resistance estimated from a female‐only training sample.

**Conclusion:**

Resistance to AD‐related neocortical tau burden differs by sex on multiple levels and is systematically sex‐biased in both its operationalization and prediction. A better understanding of sex‐specific pathways that promote resistance to neocortical tauopathy is necessary to identify protective factors that may be leveraged for targeted interventions.